# Ethanol Extract from Ampelopsis sinica Root Exerts Anti-Hepatitis B Virus Activity via Inhibition of p53 Pathway *In Vitro*

**DOI:** 10.1093/ecam/neq011

**Published:** 2011-01-09

**Authors:** Ran Pang, Jun-Yan Tao, Shu-Ling Zhang, Ke-Li Chen, Lei Zhao, Xin Yue, Yue-Feng Wang, Pian Ye, Ying Zhu, Jian-Guo Wu

**Affiliations:** ^1^Department of Hepatology and Infectious Disease, Union Hospital, Tongji Medical College, Huazhong University of Science and Technology, Wuhan 430022, China; ^2^State Key Laboratory of Virology, College of Life Sciences, Wuhan University, Wuhan, China; ^3^Faculty of Pharmacy, Hubei College of Traditional Chinese Medicine, China

## Abstract

*Ampelopsis sinica* root is widely used in Chinese folk medicine for treating liver disorders caused by the hepatitis B virus (HBV). The present study was performed in order to investigate the anti-HBV activity and mechanisms of the ethanol extract from *A. sinica* root (EASR) *in vitro.* The antiviral activity of EASR was examined by detecting the levels of HBsAg, HBeAg and extracellular HBV DNAs in stable HBV-producing human hepatoblastoma HepG2 2.2.15 cells. We found that EASR effectively suppressed the secretion of HBsAg and HBeAg from HepG2 2.2.15 cells in a dose-dependent manner, and it also suppressed the amount of extracellular HBV DNA. After EASR treatment, the percentage of apoptotic cells was found to be significantly higher than that of control by flow cytometric analysis. A luciferase reporter gene assay was used to determine the effects of EASR on the activities of HBV promoters and intracellular signaling pathways. The results showed that EASR selectively inhibited the activities of HBV promoters (Cp, S1p and Fp) and the p53 signaling pathway in HepG2 cells significantly. These data indicate that EASR exerts anti-HBV effects via inhibition of HBV promoters and the p53-associated signaling pathway, which helps to elucidate the mechanism underlying the potential therapeutic value of EASR.

## 1. Introduction

The hepatitis B virus (HBV), a member of the Hepadnaviridae family, contains a partial double-stranded circular DNA genome of 3.2 kb. Its genome has a compact organization, with four overlapping reading frames running in one direction and no non-coding regions. This unconventional genome structure reflects the unconventional mode of its replication, which involves reverse transcription of an RNA pregenome of 3.5 kb as a first step [1]. The promoters of HBV and two enhancers play important roles in the regulation of viral gene transcription.

Despite the availability of an effective vaccine, the HBV infection remains a serious global health problem. Chronic infection of HBV can result in cirrhosis and hepatocellular carcinoma (HCC), either of which can lead to a liver-related death [2]. At present, several antiviral drugs, including IFN-*α* and nucleotide analogs, are used to treat chronic hepatitis B. Nevertheless, their efficacies and serious side effects are still unsatisfactory [3]. In light of these facts, it is evident that searching for novel effective antiviral agents is an important undertaking.

Chinese medicinal herbs have been used to treat liver disease for centuries [4, 5]. *Ampelopsis*, a member of the Vitaceae family, which is distributed in tropical and subtropical regions, is used for treating liver disorders caused by HBV in Chinese folk medicine. Previous studies documented that the extract of *Ampelopsis* has hepatoprotective activity, antioxidative activity, and so forth [6–9]. However, the anti-HBV activity of the extract of *Ampelopsis* has not been investigated. In this study, we investigated the antiviral activity and mechanisms of the ethanol extract from *Ampelopsis sinica* root (EASR) *in vitro*.

## 2. Methods

### 2.1. EASR and 3TC

*Ampelopsis sinica* root was collected in August 2006 from Macheng County, Huhei Province, China. Specimens of the plant were stored in the herbarium, Hubei College of Chinese Traditional Medicine, China. The EASR used in this study was supplied by the Faculty of pharmacy, Hubei College of Traditional Chinese Medicine, China, and the extraction procedure has been previously described [6]. Before beginning the experiment, the EASR was dissolved in distilled water and then diluted with culture medium to the desired working concentration. Lamivudine (3TC), obtained from GlaxoSmithKline (Research Triangle Park, NC, USA), was used as the positive control.

### 2.2. Cell Cultures

HepG2 and HepG2 2.2.15 were procured from China Center for Typical Culture Collection (CCTCC) (Wuhan, China). Cells were cultured at 37°C in a humidified 5% CO_2_ atmosphere in Dulbecco's modified Eagle's medium (DMEM) supplemented with 10% (vol/vol) FBS, 100 U mL^−1^ penicillin G, 100 *μ*g mL^−1^ streptomycin (GIBCO, Grand Island, NY, USA). Cells were subcultured every 3 days by detaching the cells with pancreatin (0.5 mg mL^−1^). The medium was changed on the following day.

### 2.3. Cytotoxicity Assays

Cells were seeded in 96-well culture plates at a density of 1 × 10^4^ cells per well and cultured at 37°C for 24 h. Then the culture medium was removed and replaced with fresh medium supplemented with various concentrations of EASR every other day. After 9 days of culture, the cytotoxic effect of EASR was evaluated by MTT assay. Four hours prior to termination of the cultures, 20 *μ*L MTT (5 mg mL^−1^ in a phosphate buffered saline, pH 7.4) was added to the monolayer of cells. After incubation at 37°C for 4 h, 150 *μ*L DMSO was added to each well to solubilize the formazan. The optical density (OD) at 490 nm was measured by using an automatic plate reader (BIOTek, Elx800).

### 2.4. Determination of HBsAg and HBeAg

The HepG2 2.2.15 cells were plated at a density of 1 × 10^4^ cells per well on 96-well cell culture plates and were routinely cultured. Different concentrations of EASR were supplemented to the medium in triplicate 48 h after cells were plated. After incubation with EASR for 3, 5, 7 and 9 days, the supernatants were collected. The samples were centrifuged at 5000 r min^−1^ for 10 min to drop cellular debris, and then immediately used for HBsAg or HBeAg assay. The concentrations of HBsAg and HBeAg were quantified by commercial ELISA kit (Kehua Bio-engineering Corporation, Shanghai, China) according to the manufacturer's protocol. Data were calculated as percentage of control by the formula: (% of control) = (ODT)/(ODC) × 100%, where ODT and ODC indicated the cell number adjusted OD of the test drugs and the control, respectively.

### 2.5. Determination of HBV DNA

The quantity of extracellular HBV DNA in the supernatant was detected by real-time PCR (ABI PRISM 7300 Sequence Detector, PE Biosystems) based on the TaqMan technology. Viral DNA was extracted from the culture supernatant and the amount of hepatitis B viral DNA was quantified using a diagnostic kit (DaAn Gene Co. Ltd., Guangzhou, China). A series dilution of known amounts of HBV DNA was used as a control. The cycling program was: 93°C for 2 min, 10 cycles of 93°C for 45 s and 55°C for 60 s, 30 cycles of 93°C for 30 s and 55°C for 45 s.

### 2.6. Annexin V/Propidium Iodide Staining for Apoptotic Cells

The HepG2 2.2.15 cells were plated at a density of 3 × 10^5^ cells per well on six-well cell culture plates and were routinely cultured. EASR (40 *μ*g mL^−1^) was supplemented to the medium in triplicate 48 h after cells were plated. After incubation with EASR for 48 h, the cells were harvested. Cells were then washed and stained with annexin V-FITC/propidium iodide (PI) as directed by the apoptosis detection kit (Kaiji Biotechnology Company, Nanjing, China). Stained cells were kept at 4°C and protected from light until analysis on the flow cytometer.

### 2.7. Plasmid Constructions and HBV Promoter Luciferase Reporter Assay

There are five HBV promoters concerned in our study—Core promoter (Cp) (nucleotides (nt) 1603–1819 on GenBank accession no. U95551), S1 promoter (S1p) (nt 2700–2830), S2 promoter (S2p) (nt 2950–3174), X promoter (Xp) (nt 935–1361) and full-length promoter (Fp) (nt 123–1875) [10, 11]. The promoters were amplified by PCR from HepG2 2.2.15 cell genomes that contain HBV genome (U95551, ayw subtype). To generate pCp-Luc, pS1p-Luc, pS2p-Luc, pXp-Luc and pFp-Luc, the promoter regions of HBV were cloned upstream of the luciferase reporter gene of pGL3-basic (Promega, USA), respectively. The HepG2 cells were transiently transfected with the reporter vector using Sofast transfection reagent (Xiamen Sunma Biotechnology, China). After 8 h of transfection, cells were treated with 40 *μ*g mL^−1^ EASR for 48 h. The transfected cells were collected and lysed in order to perform a luciferase activity assay. HBV promoter activities were determined by measuring luciferase activity in a TD-20/20 luminometer (Turner BioSystems, USA) using the Luciferase Reporter Assay System (Promega, USA).

### 2.8. Intracellular Signal Transduction Pathway Luciferase Reporter Assay

PathDetect Cis-/Trans-Reporting Systems (pNF-*κ*B-Luc, pAP-1-Luc, pISRE-Luc, p53-Luc, pFA2-Elk1, pFA2-cJun, pFA2-CHOP and pFR-Luc) were obtained from Stratagene (CA, USA). HepG2 cells were transiently transfected with pNF*κ*B-Luc, pAP-1-Luc, pISRE-Luc, p53-Luc, pFA2-Elk1 plus pFR-Luc, pFA2-cJun plus pFR-Luc and pFA2-CHOP plus pFR-Luc using the Sofast transfection reagent, respectively. After 8 h of transfection, cells were treated with 40 *μ*g mL^−1^ EASR for 48 h. The activity of each signaling pathway was determined by measuring the luciferase activities of the reporter in treated and untreated transfectants.

### 2.9. Statistical Analysis

Statistical analysis was performed using the SPSS 12.0 software (SPSS Inc., Chicago, IL, USA). Data were expressed as means ± SD. Student's *t*-test and one-way ANOVA were used to determine the statistical significance of differences between the test samples and control. A *P* < .05 was considered statistically significant.

## 3. Results

### 3.1. Cytotoxicity of EASR

**Figure 1:**
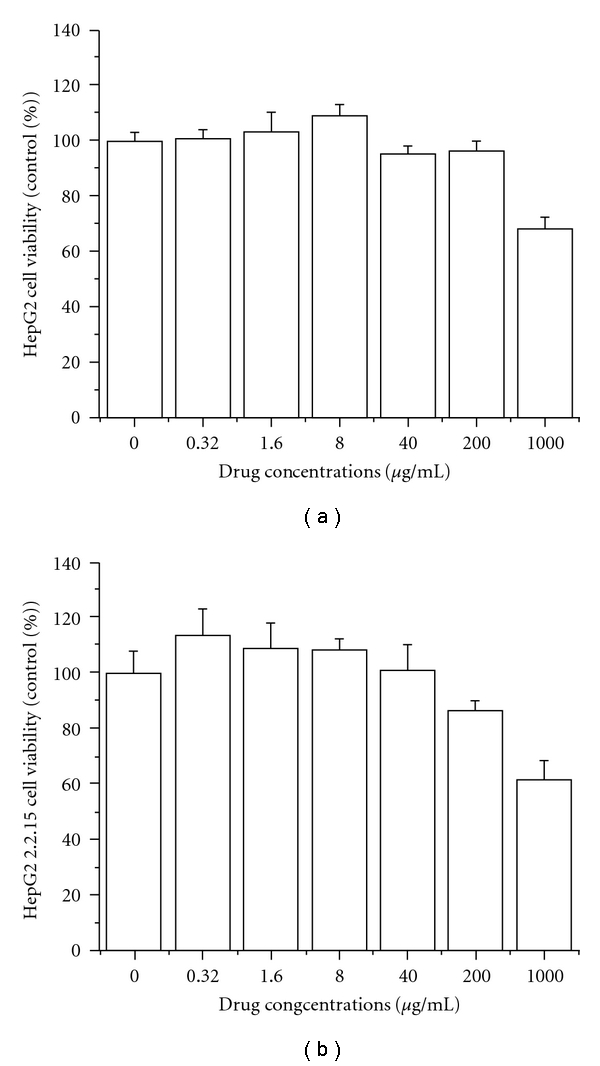
The cytotoxicity of EASR on HepG2 and HepG2 2.2.15 cells. The cell viability was evaluated by MTT assay. The data are presented as means ± SD (*n* = 3). (a) HepG2, and (b) HepG2 2.2.15 cells.

The cytotoxicity of EASR on the cell viability of HepG2 2.2.15 cells and HepG2 cells was evaluated by using the MTT assay. As shown in Figure 1, there was no significant difference of cell viability between EASR-treated groups whose concentrations were <200 *μ*g mL^−1^ and the control group. But higher concentrations of EASR were demonstrated to be cytotoxic.

### 3.2. Anti-HBV Activity of EASR

**Figure 2:**
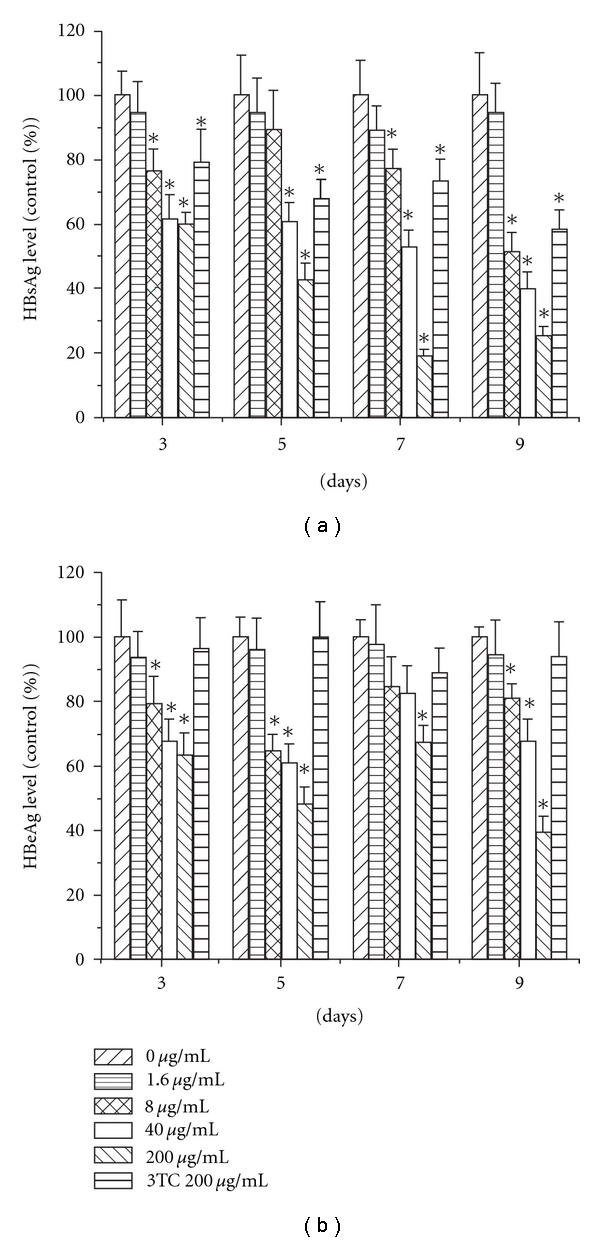
The effect of EASR on HBsAg and HBeAg secretion in vitro. The cells were cultured with different concentrations of EASR (1.6, 8, 40 and 200 mg mL^−1^) or 3TC at 200 mg mL^−1^ for 3, 5, 7 and 9 days. The concentrations of HBsAg and HBeAg in culture supernatants were quantified by ELISA assay. The results revealed that EASR could inhibit HBsAg and HBeAg secretion dose dependently in HepG2 2.2.15 cells. The data are presented as means ± SD (*n* = 3). *P < .05 as compared with the no drug group. (a) HBsAg and (b) HBeAg.

The HBsAg and HBeAg in the supernatant were determined by ELISA assay. The results indicated that EASR could inhibit the secretion of HBsAg and HBeAg dose dependently in HepG2 2.2.15 cells (*P* < .05; Figure 2), and 3TC has little effect on the HBeAg secretion. EASR was more potent than 3TC for inhibiting the HBsAg and HBeAg secreted by HepG2 2.2.15 cells.

**Figure 3:**
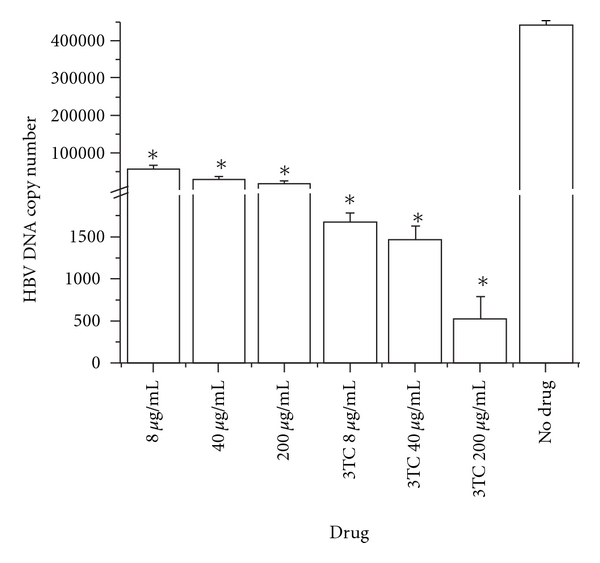
The effect of EASR on HBV DNA replication. Extracellular HBV DNA was isolated from the culture supernatant at 9 days and quantified by real-time PCR. The results indicated that EASR could inhibit HBV DNA replication dose dependently *in vitro*. The data are presented as means ± SD (*n* = 3). *P < .05 as compared with the no drug group.

To further confirm the antiviral activity of EASR in HepG2 2.2.15 cells, the extracellular HBV DNA levels were evaluated by real-time PCR. Consistent with the inhibitory effects on HBsAg and HBeAg secretion, EASR treatment of HepG2 2.2.15 cells at various concentrations resulted in the reduction of the extracellular HBV DNA levels in a dose-dependent manner (*P* < .05; Figure 3).

### 3.3. Induction of Apoptosis by EASR

**Figure 4:**
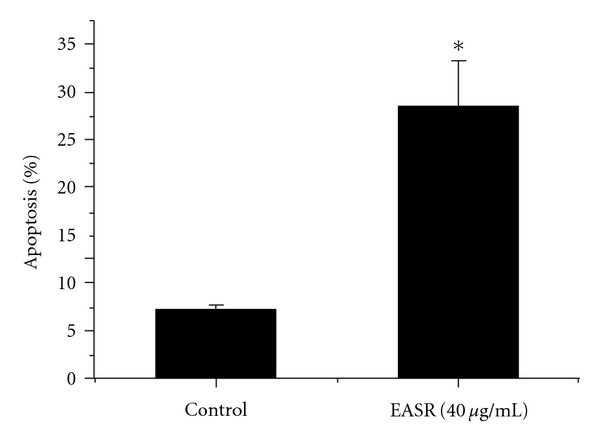
The apoptosis of HepG2 2.2.15 cells after EASR treatment. The results indicated that the percentage of apoptotic cells increased significantly following incubation with EASR (40 *μ*g mL^−1^) for 48 h. The data are presented as means ± SD (*n* = 3). *P < .05 as compared with the no drug group.

Flow cytometric analysis was performed to assess the apoptosis of HepG2 2.2.15 cells after 48 h of exposure to EASR. As shown in Figure 4, the percentage of apoptotic cells increased significantly following EASR treatment (*P* < .05).

### 3.4. Inhibition of HBV Promoter Activities by EASR

**Figure 5:**
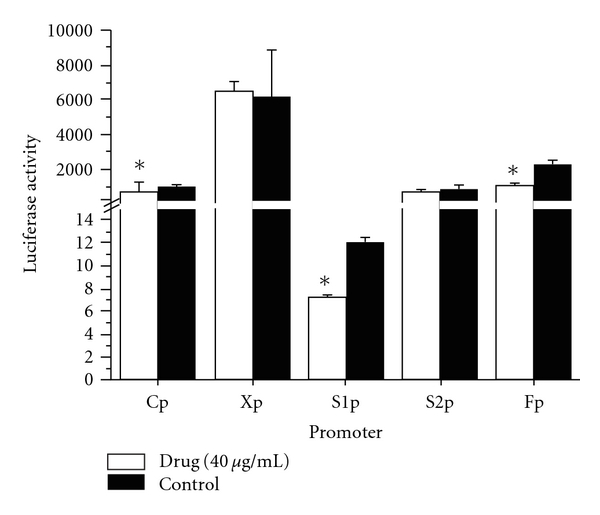
The effect of EASR on HBV transcription *in vitro*. The plasmids of the five HBV promoters (Cp, S1p, S2p, Xp and Fp) were transfected into HepG2 cells, respectively. In the experimental group, cells were treated with 40 *μ*g mL^−1^ EASR for 48 h. And the control group was cultured without EASR treatment. HBV promoter activities were then determined by luciferase reporter assay. EASR selectively inhibited the activities of HBV promoters (Cp, S1p and Fp) in HepG2 cells significantly (*P* < .05). The data are presented as means ± SD (*n* = 3). *P < .05 as compared with the control group.

To examine the effect of EASR on HBV promoter activities, we constructed five plasmids (pCp-Luc, pS1p-Luc, pS2p-Luc, pXp-Luc and pFp-Luc) containing the different promoter regions of HBV followed by the luciferase reporter gene. After transient transfection of these plasmids into HepG2 cells and EASR treatment, the viral promoter activities were examined by a luciferase reporter assay. As shown in Figure 5, EASR inhibited the activities of HBV promoters (Cp, S1p and Fp) in HepG2 cells significantly (*P* < .05).

### 3.5. Inhibition of the p53 Signaling Pathway by EASR

**Figure 6:**
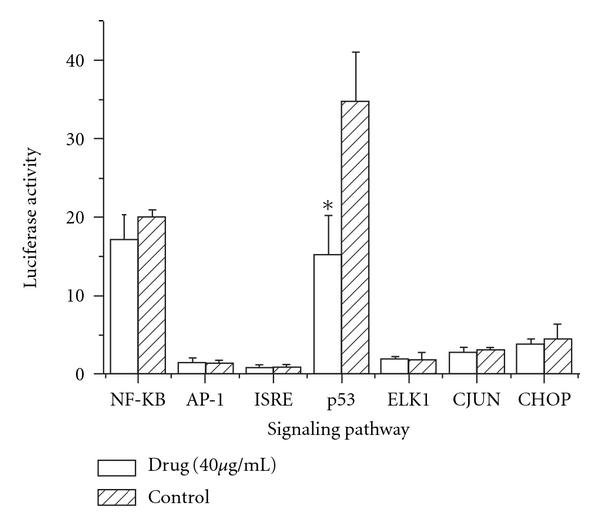
The effect of EASR on cell signaling pathway activity *in vitro*. The HepG2 cells were transfected with various plasmids provided by PathDetect Cis-/Trans-Reporting Systems. In the experimental group, cells were treated with 40 *μ*g mL^−1^ EASR for 48 h. And the control group was cultured without EASR treatment. The signaling pathway activities were determined by luciferase reporter assay. EASR inhibited the activity of p53 pathway significantly (*P* < .05). The data are presented as means ± SD (*n* = 3). *P < .05 as compared with the control group.

To determine the effect of EASR on signaling pathway activities, a series of plasmids containing the luciferase reporter gene were transfected into HepG2 cells. After transfection, we examined the signaling pathway activities by luciferase assay. As shown in Figure 6, EASR selectively inhibited the activity of p53-associated signaling pathway significantly (*P* < .05).

## 4. Discussion

Although several pharmacological strategies are currently being implemented to treat affected patients, no satisfactory antiviral therapy against HBV infection has yet been fully developed. Thus, it has become urgent to find new and effective anti-HBV drugs. *Ampelopsis*, a plant used in folk medicine to treat liver disease, is widely distributed in tropical and subtropical regions [7]. The active ingredients of *Ampelopsis* are mainly flavonoids, such as ampelopsin, dihydromyricetin, myricetin, and so forth [12]. The flavonoids have recently aroused considerable interest because of their potential beneficial effects on human health; they have been reported to have antiviral, anti-allergic, anti-platelet, anti-inflammatory, antitumor and anti-oxidant activities [13].

In the present study, we investigated the anti-HBV activity of EASR in stably HBV-transfected HepG2 2.2.15 cells, which can continuously produce complete virion particles of HBV and a high level of viral proteins [14]. We found that EASR could decrease the extracellular HBV DNA levels and the secretion of HBsAg and HBeAg in a dose-dependent manner. These results demonstrated for the first time that EASR possesses potent inhibitory activity against HBV gene expression and replication *in vitro*. And 3TC was found to have no obvious inhibitory effects on HBeAg secretion. The result was similar to those from other experiments [15, 16]. HBeAg plays an important role in viral persistence, and the presence of HBeAg in serum indicates active viral replication in hepatocytes [17]. Several prospective studies have uniformly shown that high HBeAg and HBV DNA levels are associated with an increased risk of liver fibrosis, cirrhosis and HCC [18–20]. The HBV viral load is an useful prognostic parameter to evaluate the extent of liver disease in patients with chronic HBV infection [21]. To determine whether EASR treatment could decrease the liver damage resulting from HBV infection, further work is needed to investigate the antiviral activity of EASR *in vivo*.

The precise modulation of HBV gene expression is essential for replication of the virus, and the expression of HBV is mainly regulated at the transcription initiation level [22]. To identify whether the *cis*-element in HBV genome is responsible for the anti-HBV activity of EASR, we examined the effect of EASR on the activities of five different HBV promoters: Cp, S1p, S2p, Xp and Fp. When linked to luciferase reporter genes, the activity of the individual HBV promoters was determined by transfection experiments. Our studies revealed that EASR is a potent transcriptional inhibitor of Cp, S1p and Fp, but has no effect on S2p and Xp activities in human hepatoma cells. Cp plays a central role in HBV gene expression and replication, directing the transcription of pregenomic RNA and precore mRNA [23]. The S1p is indispensable for the formation of the transcription initiation complex [10]. The full-length promoter has a whole region of the HBV genome (U95551, ayw subtype) between nt 123 and 1875, covering the intact regions of enhancer I, Xp, Cp and enhancer II [11]. These promoters of HBV may act as molecular switches, determining the gene activity. The suppression of these “switches" could further influence the transcription and translation of HBV genome, resulting in the overall inhibition of viral replication. Two enhancers (Enhancers I and II) also play important roles in the regulation of viral gene transcription [10]. Either of the two enhancers could activate the HBV promoters *in vitro* [24]. But exactly how EASR inhibits HBV viral Cp, S1p and Fp activities is still unknown.

To further investigate the molecular mechanism of anti-HBV activity of EASR, we examined the influence of EASR on several well-defined intracellular signaling pathways by use of a luciferase reporter assay. A series of plasmids containing the luciferase reporter gene were transfected into human hepatoma cells to analyze the induction of the intracellular signal pathways after EASR treatment. We found EASR selectively inhibited the activity of the p53-associated pathway. Previous studies have demonstrated that p53 plays a pivotal role in the modulation of cell-cycle arrest, cell differentiation and induction of apoptosis [25–27]. p53 is also essential for a host's antiviral innate immune responses [28]. It has been reported to associate with the replication of several viruses, but its role in antiviral defense is conflicting. p53 enhances the replications of adenovirus, cytomegalovirus, encephalomyocarditis virus, human parainfluenza virus and respiratory syncytial virus, but it limits herpes simplex virus, poliovirus, hepatitis C virus and vesicular stomatitis virus replications [29–35]. p53 causes G1 phase cell-cycle arrest that protects cells from virus-mediated cell death [31]. Down-regulatation of p53 could result in a decrease in G1 arrest and induce apoptosis, thereby limiting viral replication [29]. Our results revealed that EASR could induce apoptosis via inhibitions of p53 pathway. We propose that the effect of p53 on viral replication is also dependent on the replicative cycle of the virus.

**Figure 7:**
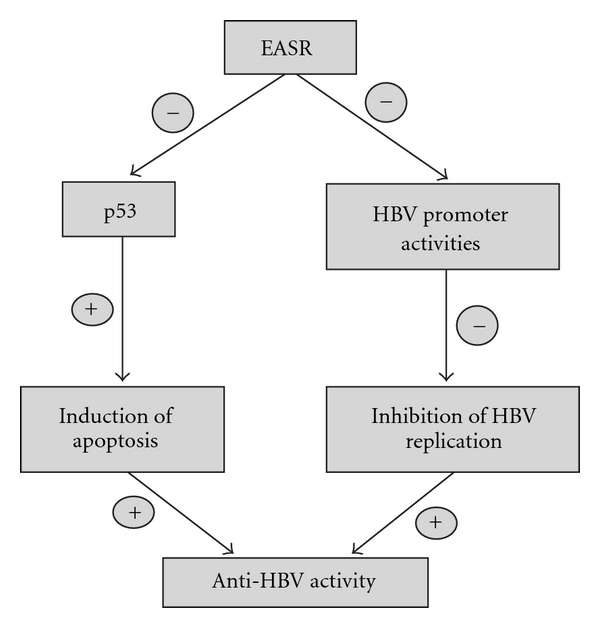
EASR exerts anti-HBV effects via inhibition of HBV transcription and the p53-associated signaling pathway.

In conclusion, EASR could exert anti-HBV activity by decreasing both the level of extracellular HBV DNA and the secretion of HBsAg and HBeAg antigens. We also found the antiviral activity of EASR is associated with the *in vitro* inhibitions of HBV promoters (Cp, S1p and Fp) and p53 pathway. These results indicate that EASR exerts anti-HBV effects via inhibition of HBV transcription and the p53-associated signaling pathway (Figure 7). This helps to elucidate the mechanism underlying the potential therapeutic value of EASR.
