# Corrigendum to “Ethanol Extract from Ampelopsis sinica Root Exerts Anti-Hepatitis B Virus Activity via Inhibition of p53 Pathway *In Vitro*”

**DOI:** 10.1155/2015/603232

**Published:** 2015-08-19

**Authors:** Ran Pang, Jun-Yan Tao, Shu-Ling Zhang, Ke-Li Chen, Lei Zhao, Xin Yue, Yue-Feng Wang, Pian Ye, Ji-Hua Dong, Ying Zhu, Jian-Guo Wu

**Affiliations:** ^1^Department of Hepatology and Infectious Disease, Union Hospital, Tongji Medical College, Huazhong University of Science and Technology, Wuhan 430022, China; ^2^State Key Laboratory of Virology, College of Life Sciences, Wuhan University, Wuhan, China; ^3^Faculty of Pharmacy, Hubei College of Traditional Chinese Medicine, China; ^4^Central Lab, Union Hospital, Tongji Medical College, Huazhong University of Science and Technology, Wuhan, China

In the paper titled “Ethanol Extract from Ampelopsis sinica Root Exerts Anti-Hepatitis B Virus Activity via Inhibition of p53 Pathway* In Vitro*” [1], the words “Ran Pang and Jun-Yan Tao contribute equally to this work” should be added to the footnote. Dr. Ji-Hua Dong should be included in the author's list, because he has contributed to the scientific content of the paper. Thus, the affiliation “*Central Lab, Union Hospital, Tongji Medical College, Huazhong University of Science and Technology, Wuhan, China*” will be added as a new address as above.
